# Active HPV infection and its influence on survival in head and neck squamous-cell cancer

**DOI:** 10.1007/s00432-020-03218-6

**Published:** 2020-05-05

**Authors:** Anna Janecka-Widła, Anna Mucha-Małecka, Kaja Majchrzyk, Krzysztof Halaszka, Marcin Przewoźnik, Dorota Słonina, Beata Biesaga

**Affiliations:** 1 Department of Tumour Pathology, Maria Sklodowska-Curie Institute - Oncology Center, Cracow Branch, Cracow, Poland; 2 Department of Radiotherapy, Maria Sklodowska-Curie Institute - Oncology Center, Cracow Branch, Cracow, Poland; 3 Center for Translational Research and Molecular Biology of Cancer, Maria Sklodowska-Curie Institute - Oncology Center, Gliwice Branch, Gliwice, Poland

**Keywords:** HPV, Active infection, HNSCC, p16 overexpression, Nested PCR, Survival

## Abstract

**Purpose:**

HPV is involved in the development of some head and neck squamous-cell carcinomas (HNSCC). It was suggested that only transcriptionally active virus can induce carcinogenesis, therefore, the aim of our study was to analyze the frequency of active HPV infection, virus type, and its prognostic role in HNSCC patients.

**Methods:**

Status of active HPV infection was assessed for 155 HNSCC patients based on p16 expression and HPV DNA presence. Univariate and multivariate analyses with Cox proportional regression model were performed to select independent prognostic factors.

**Results:**

Active HPV infection was detected in 20.65% of patients. We identified 16.0, 40.9 and 1.7% of HPV positive oral cavity, oropharyngeal, and laryngeal cancer cases, respectively. HPV16 was dominant (81.25%) followed by HPV35 (9.38%) and double infections with HPV16 and 35 (6.25%) or HPV35 and 18 (3.12%). Patients with active HPV infection demonstrated significantly higher survival than HPV negative ones (OS 80.89% vs. 37.08%, *p* = 0.000; DFS 93.0% vs. 53.35%, *p* = 0.000, respectively). Longer OS and DFS were maintained for infected patients when oropharyngeal and non-oropharyngeal cases were analyzed separately. Interestingly, all patients infected with other than HPV16 types survived 5 years without cancer progression. In the analyzed group of 155 patients the strongest independent favourable prognostic factor for both OS and DFS was HPV presence.

**Conclusions:**

High prevalence of HPV-driven HNSCC (mostly within oropharynx) was detected, with HPV16 type the most frequent, followed by HPV35 and HPV18. The presence of active HPV infection improved survival of both oropharyngeal and non-oropharyngeal cancer patients and should be taken into account in treatment planning.

## Introduction

Human papillomavirus (HPV) is a small, double-stranded DNA virus responsible for the most frequent sexually transmitted viral infection worldwide, which causes an infection of squamous epithelium in the human body. There are more than 100 types of HPV classified into low-risk or high-risk (oncogenic) group. Low-risk HPVs (mostly HPV6 and 11) can cause benign and transient lesions. On the other hand, high-risk HPV infection may stimulate malignant transformation and HPV16, 18, 31, 33, 35, 39, 45, 51, 52, 56, 58, 59 and 68 belong to this group (de Martel et al. 2017).

HPV role has been well established in cervical cancer, however, virus has been also detected in other anogenital cancers such as vulvar (Zhang et al. 2018), vaginal (Sinno et al. 2014), anal (Ravenda et al. 2014) and penile (Rubin et al. 2001). Moreover, there is growing evidence that HPV may be involved in the development of some head and neck squamous-cell carcinomas (HNSCC). Wang et al. (2013) emphasized that HPV-derived HNSCC is a disease highly different than classical HNSCC (associated with exposure to tobacco or alcohol) with distinct aetiology, epidemiological and molecular characteristics as well as treatment response and survival.

It was suggested that only transcriptionally active HPV can induce carcinogenesis. When viral DNA integrates into the host cell genome, E6 and E7 oncoproteins become overexpressed and such deregulated overexpression allows (via the inactivation of p53 and pRb) uncontrolled cell growth without checkpoint controls, that further leads to accumulation of mutations, cell transformation and finally causes cancer (Ganguly and Parihar 2009; Narisawa-Saito and Kiyono 2007).

The data on HPV prevalence in HNSCC specimens are highly heterogeneous so it is still the subject of worldwide discussion. According to the literature HPV positive tissues represented 0 (Albano et al. 2017) to 44% (Duray et al. 2012) of oral cavity, 0 (Albano et al. 2017) to 86% (D’Souza et al. 2016) of oropharyngeal, 0–22.2% (Hauck et al. 2015) of hypopharyngeal and 0 (Onerci Celebi et al. 2018) to 35.7% (Dahlstrom et al. 2003) of laryngeal tumour specimens. Such huge discrepancies may resulted from differences in geographic locations, time period of patients’ recruitment and methodology (different qualification criteria, demographic groups, number of cases, cut-off points, HPV detection methods, limited spectrum of HPV types analyzed).

There are many HPV detection methods in head and neck cancer patients and each test has its own strengths and weaknesses (see Venuti and Paolini 2012). Detection of E6/E7 mRNA in fresh frozen cancer samples was suggested to be a gold standard for identification of HPV presence (Smeets et al. 2007; Bussu et al. 2019), because it exactly reflects an active viral infection. However, in most studies other methods are used such as immunohistochemical p16 staining or HPV DNA detection, but neither of these tests alone is optimal for HPV status identification (they generate false positive results) (own observations; Smeets et al. 2007; Dalianis et al. 2015). HPV DNA reflects the status of existing infection but does not indicate whether HPV is transcriptionally active or not. P16, in turn, is a surrogate marker indicating active HPV infection, but its overexpression may not exactly match the HPV DNA, because it may be also caused by other, non-viral factors. Hence, it was suggested to improve HPV detection accuracy by combining these two methods, i.e. using p16 staining followed by HPV DNA PCR analysis (Smeets et al. 2007; Wang et al. 2013; Golusiński et al. 2012), since this algorithm allowed to obtain 100% sensitivity and specificity with HPV E6/E7 mRNA detection (Smeets et al. 2007).

Therefore, in this study we decided to analyze the frequency of active HPV infection (based on PCR and immunohistochemical p16 staining), virus type and its prognostic role in patients with HNSCC from South-Central Poland.

## Materials and methods

### Patients

A group of 155 HNSCC patients from South-Central Poland, treated between 1991 and 2014, was enrolled in this study. There were 25 oral cavity, 66 oropharyngeal, 6 hypopharyngeal and 58 laryngeal cancer cases. For all participants, levels of smoking (number of cigarettes per day x years of smoking) and alcohol drinking (‘low’—for no or occasional alcohol drinkers or ‘high’—for alcoholics and people drink more than 15 drinks of high percentage alcohol per week) as well as treatment outcome (alive without cancer symptoms; cancer progression: treatment failure, local recurrence or distant metastasis; death from other reasons than cancer disease) were noted. Patients and tumours detailed characteristics were summarized in Table 1 (all HNSCC patients) and Table 2 (oropharyngeal patients only).

**Table 1:** Clinical and histopathological features in relation to active HPV infection

Feature	All N (%)^a^	HPV + N (%)^a^	HPV- N (%)^a^	*p*-value (*χ*^2^ test)^d^	All N (%)^c^	HPV16 + N (%)	HPV- N (%)	*p*-value (*χ*^2^ test)^d^
All	155 (100)	32 (20.65)	123 (79.35)		151 (100)	28 (18.54)	123 (81.46)	
Age								
≤ 52 years	51 (32.90)	6 (18.75)	45 (36.59)	0.056	50 (33.11)	5 (17.86)	45 (36.59)	0.057
> 52 years	104 (67.10)	26 (81.25)	78 (63.41)		101 (66.89)	23 (82.14)	78 (63.41)	
Gender								
Male	130 (83.87)	21 (65.63)	109 (88.62)	**0.002**	128 (84.77)	19 (67.86)	109 (88.62)	**0.006**
Female	25 (16.13)	11 (34.37)	14 (11.38)		23 (15.23)	9 (32.14)	14 (11.38)	
Performance status in the Karnofsky scale								
≤ 80%	90 (58.06)	12 (37.50)	78 (63.41)	**0.008**	89 (58.94)	11 (39.29)	78 (63.41)	**0.019**
> 80%	65 (41.94)	20 (62.50)	45 (36.59)		62 (41.06)	17 (60.71)	45 (36.59)	
Tumour site								
Oral cavity	25 (16.13)	4 (12.50)	21 (17.07)	**0.000**	25 (16.56)	4 (14.29)	21 (17.07)	**0.000**
Oropharynx	66 (42.58)	27 (84.37)	39 (31.71)		62 (41.06)	23 (82.14)	39 (31.71)	
Hypopharynx	6 (3.87)	0 (0.0)	6 (4.88)		6 (3.97)	0 (0.0)	6 (4.88)	
Larynx	58 (37.42)	1 (3.13)	57 (46.34)		58 (38.41)	1 (3.57)	57 (46.34)	
*T* stage								
1	2 (1.29)	0 (0.0)	2 (1.63)	**0.041**	2 (1.32)	0 (0.0)	2 (1.63)	**0.039**
2	27 (17.42)	9 (28.12)	18 (14.63)		26 (17.22)	8 (28.57)	18 (14.63)	
3	78 (50.32)	19 (59.38)	59 (47.97)		76 (50.33)	17 (60.72)	59 (47.97)	
4	48 (30.97)	4 (12.50)	44 (35.77)		47 (31.13)	3 (10.71)	44 (35.77)	
*N* stage								
0	32 (20.65)	2 (6.25)	30 (24.39)	0.132	32 (21.19)	2 (7.14)	30 (24.39)	0.228
1	28 (18.06)	8 (25.00)	20 (16.26)		25 (16.56)	5 (17.86)	20 (16.26)	
2	84 (54.19)	19 (59.38)	65 (52.85)		83 (54.97)	18 (64.29)	65 (52.85)	
3	11 (7.10)	3 (9.37)	8 (6.50)		11 (7.28)	3 (10.71)	8 (6.50)	
Grade								
1	48 (30.97)	10 (31.25)	38 (30.89)	0.329	48 (31.79)	10 (35.71)	38 (30.89)	0.462
2	85 (54.84)	20 (62.50)	65 (52.85)		81 (53.64)	16 (57.15)	65 (52.85)	
3	22 (14.19)	2 (6.25)	20 (16.26)		22 (14.57)	2 (7.14)	20 (16.26)	
Keratinization								
No	64 (41.29)	21 (65.63)	43 (34.96)	**0.002**	60 (39.74)	17 (60.71)	43 (34.96)	**0.012**
Yes	91 (58.71)	11 (34.37)	80 (65.04)		91 (60.26)	11 (3.29)	80 (65.04)	
The level of smoking^b^								
≤ 200	33 (21.29)	16 (50.00)	17 (13.82)	**0.000**	31 (20.53)	14 (50.00)	17 (13.82)	**0.000**
> 200	122 (78.71)	16 (50.00)	106 (86.18)		120 (79.47)	14 (50.00)	106 (86.18)	
The level of drinking								
Low	67 (43.23)	23 (71.88)	44 (35.77)	**0.000**	64 (42.38)	20 (71.43)	44 (35.77)	**0.001**
High	88 (56.77)	9 (28.12)	79 (64.23)		87 (57.62)	8 (28.57)	79 (64.23)	
Treatment								
Definitive CRT	31 (20.00)	13 (40.63)	18 (14.63)	**0.000**	29 (19.21)	11 (39.29)	18 (14.63)	**0.000**
Surgery + CRT	10 (6.45)	6 (18.75)	4 (3.25)		9 (5.95)	5 (17.86)	4 (3.25)	
Definitive RT	12 (7.74)	2 (6.25)	10 (8.13)		12 (7.95)	2 (7.13)	10 (8.13)	
Surgery + RT	76 (49.04)	6 (18.75)	70 (56.92)		76 (50.33)	6 (21.43)	70 (56.92)	
Induction CT	26 (16.77)	5 (15.62)	21 (17.07)		25 (16.56)	4 (14.29)	21 (17.07)	
Treatment outcome								
Alive at the last follow-up	61 (39.35)	24 (75.00)	37 (30.08)	**0.000**	58 (38.41)	21 (75.00)	37 (30.08)	**0.001**
Treatment failure	7 (4.52)	1 (3.13)	6 (4.87)		7 (4.63)	1 (3.57)	6 (4.87)	
Local recurrence	34 (21.94)	2 (6.24)	32 (26.02)		34 (22.52)	2 (7.14)	32 (26.02)	
Distant metastases	16 (10.32)	1 (3.13)	15 (12.20)		16 (10.60)	1 (3.57)	15 (12.20)	
Death from other reasons	37 (23.87)	4 (12.50)	33 (26.83)		36 (23.84)	3 (10.72)	33 (26.83)	

^a^Column percentage

^b^Number of cigarettes per day x years of smoking

^c^151 patients (patients infected with other than HPV16 types were excluded)

^d^Values in bold indicate statistically significant differences between groups at the *p* < 0.05 level

**Table 2:** Clinical and histopathological features in relation to active HPV infection in 66 oropharyngeal cancer patients

Feature	All N (%)^a^	HPV + N (%)^a^	HPV- N (%)^a^	*p*-value (*χ*^2^ test)^d^	All N (%)^c^	HPV16 + N (%)	HPV- N (%)	*p*-value (*χ*^2^ test)^d^
All	66 (100)	27 (40.91)	39 (59.09)		62 (100)	23 (37.10)	39 (62.90)	
Age								
≤ 52 years	21 (31.82)	5 (18.52)	16 (41.03)	0.054	20 (32.26)	4 (17.39)	16 (41.03)	0.054
> 52 years	45 (68.18)	22 (81.48)	23 (58.97)		42 (67.74)	19 (82.61)	23 (58.97)	
Gender								
Male	51 (77.27)	17 (62.96)	34 (87.18)	**0.021**	49 (79.03)	15 (65.22)	34 (87.18)	**0.040**
Female	15 (22.73)	10 (37.04)	5 (12.82)		13 (20.97)	8 (34.78)	5 (12.82)	
Performance status in the Karnofsky scale								
≤ 80%	28 (42.42)	11 (40.74)	17 (43.59)	0.818	27 (43.55)	10 (43.48)	17 (43.59)	0.993
> 80%	38 (57.58)	16 (59.26)	22 (56.41)		35 (56.45)	13 (56.52)	22 (56.41)	
*T* stage								
1	0 (0.0)	0 (0.0)	0 (0.0)	0.261	0 (0.0)	0 (0.0)	0 (0.0)	0.229
2	15 (22.73)	8 (29.63)	7 (17.95)		14 (22.58)	7 (30.43)	7 (17.95)	
3	35 (53.03)	15 (55.56)	20 (51.28)		33 (53.23)	13 (56.53)	20 (51.28)	
4	16 (24.24)	4 (14.81)	12 (30.77)		15 (24.19)	3 (13.04)	12 (30.77)	
*N* stage								
0	11 (16.67)	2 (7.41)	9 (23.08)	0.188	11 (17.74)	2 (8.70)	9 (23.08)	0.464
1	13 (19.70)	8 (29.62)	5 (12.82)		10 (16.13)	5 (21.74)	5 (12.82)	
2	36 (54.55)	15 (55.56)	21 (53.85)		35 (56.45)	14 (60.86)	21 (53.85)	
3	6 (9.08)	2 (7.41)	4 (10.25)		6 (9.68)	2 (8.70)	4 (10.25)	
Grade								
1	25 (37.88)	9 (33.33)	16 (41.03)	0.278	25 (40.32)	9 (39.13)	16 (41.03)	0.497
2	35 (53.03)	17 (62.97)	18 (46.15)		31 (50.00)	13 (56.52)	18 (46.15)	
3	6 (9.09)	1 (3.70)	5 (12.82)		6 (9.68)	1 (4.35)	5 (12.82)	
Keratinization								
No	28 (42.42)	17 (62.96)	11 (28.21)	**0.005**	24 (38.71)	13 (56.52)	11 (28.21)	**0.027**
Yes	38 (57.58)	10 (37.04)	28 (71.79)		38 (61.29)	10 (43.48)	28 (71.79)	
The level of smoking^b^								
≤ 200	22 (33.33)	13 (48.15)	9 (23.08)	**0.034**	20 (32.26)	11 (47.83)	9 (23.08)	**0.044**
> 200	44 (66.67)	14 (51.85)	30 (76.92)		42 (67.74)	12 (52.17)	30 (76.92)	
The level of drinking								
Low	29 (43.94)	19 (70.37)	10 (25.64)	**0.000**	26 (41.94)	16 (69.57)	10 (25.64)	**0.001**
High	37 (56.06)	8 (29.63)	29 (74.36)		36 (58.06)	7 (30.43)	29 (74.36)	
Treatment								
Definitive CRT	22 (33.33)	13 (48.16)	9 (23.08)	0.076	20 (32.26)	11 (47.83)	9 (23.08)	0.119
Surgery + CRT	6 (9.09)	4 (14.81)	2 (5.12)		5 (8.06)	3 (13.04)	2 (5.12)	
Definitive RT	6 (9.09)	2 (7.41)	4 (10.26)		6 (9.68)	2 (8.70)	4 (10.26)	
Surgery + RT	13 (19.70)	4 (14.81)	9 (23.08)		13 (20.97)	4 (17.39)	9 (23.08)	
Induction CT	19 (28.79)	4 (14.81)	15 (38.46)		18 (29.03)	3 (13.04)	15 (38.46)	
Treatment outcome								
Alive at the last follow-up	30 (45.45)	19 (70.38)	11 (28.21)	**0.014**	27 (43.55)	16 (69.56)	11 (28.21)	**0.026**
Treatment failure	2 (3.03)	1 (3.70)	1 (2.55)		2 (3.23)	1 (4.35)	1 (2.55)	
Local recurrence	14 (21.21)	2 (7.41)	12 (30.77)		14 (22.58)	2 (8.70)	12 (30.77)	
Distant metastases	5 (7.58)	1 (3.70)	4 (10.26)		5 (8.06)	1 (4.35)	4 (10.26)	
Death from other reasons	15 (22.73)	4 (14.81)	11 (28.21)		14 (22.58)	3 (13.04)	11 (28.21)	

^a^Column percentage

^b^Number of cigarettes per day x years of smoking

^c^151 patients (patients infected with other than HPV16 types were excluded)

^d^Values in bold indicate statistically significant differences between groups at the *p* < 0.05 level

For each patient a set of available blocks with formalin-fixed paraffin-embedded (FFPE) cancer specimens obtained during surgery were collected. Histopathological reverification for each tissue was performed to confirm cancer diagnosis, assess grade and keratinization status as well as to select a block with at least 50% of tumour component for molecular analyses. The study was approved by Ethical Committee at the Regional Medical Chamber in Cracow, Poland (registration no. 109/KBL/OIL/2012).

### DNA extraction

DNA was extracted from selected FFPE tissues using ReliaPrep FFPE gDNA Miniprep System (Promega, WI, USA) according to manufacturer suggestions. The only modification introduced was to extend digestion at 56 °C from 1 h to overnight to provide improved quantity and quality of DNA (Janecka et al. 2015).

In brief, DNA was isolated from 4 µm thick 3–5 sections. To prevent cross-contamination, we used fresh, sterile microtome blade for each tissue. Deparaffinization was performed using mineral oil at 80 °C. Then, lysis buffer was added to samples and after centrifugation two phases were observed: aqueous containing tissue and oil containing dissolved paraffin. Proteinase K was added to aqueous phase and samples were incubated overnight at 56 °C. Samples were further incubated for 1 h at 80 °C, digested with RNase A and mixed with BL buffer and 96% ethanol. After centrifugation the entire aqueous phase containing DNA was transferred into the binding column. It was washed twice and finally eluted. DNA concentration and purity (measured as A260/280 and A260/230 ratios) were evaluated spectrophotometrically with Biophotometer Plus (Eppendorf, Germany). Samples were stored at − 20 °C until used.

### Nested PCR

To screen HNSCC samples for the presence of HPV DNA, PGMY09/PGMY11 (Gravitt et al. 2000) and GP5 + /GP6 + (de Roda Husman et al. 1995) primer sets were used, according to our best knowledge—for this experiment for the first time in Poland. Full list of primer sequences is shown in Table 3. Nested PCR method consisted of two successive PCR runs and the product of the first reaction with PGMY09/PGMY11 serves as a template in the second run with GP5 + /GP6 + . It was demonstrated that using PGMY/GP + primers in nested PCR improved detection of HPV DNA not only in cervical samples, where the method allowed to detect even 1 copy of HPV DNA (Fuessel Haws et al. 2004), but also in oral SCC samples (Erhart et al. 2016). The combination of mentioned primers is also more sensitive than popular MY/GP + and able to detect low copy HPVs as well as wider range of virus types, especially in cases of multiple infections (Gravitt et al. 2000; Fuessel Haws et al. 2004). They allow to amplify L1 gene fragment of multiple HPV types simultaneously, however, without indication of virus type precisely.

**Table 3:** Sequences of PCR primers

Primer	Sequence (5′—3′)
GP5 +	TTT GTT ACT GTG GTA GAT ACT AC
GP6 +	GAA AAA TAA ACT GTA AAT CAT ATT C
PGMY11-A^a^	GCA CAG GGA CAT AAC AAT GG
PGMY11-B	GCG CAG GGC CAC AAT AAT GG
PGMY11-C	GCA CAG GGA CAT AAT AAT GG
PGMY11-D	GCC CAG GGC CAC AAC AAT GG
PGMY11-E	GCT CAG GGT TTA AAC AAT GG
PGMY09-F	CGT CCC AAA GGA AAC TGA TC
PGMY09-G	CGA CCT AAA GGA AAC TGA TC
PGMY09-H	CGT CCA AAA GGA AAC TGA TC
PGMY09-I	G CCA AGG GGA AAC TGA TC
PGMY09-J	CGT CCC AAA GGA TAC TGA TC
PGMY09-K	CGT CCA AGG GGA TAC TGA TC
PGMY09-L	CGA CCT AAA GGG AAT TGA TC
PGMY09-M	CGA CCT AGT GGA AAT TGA TC
PGMY09-N	CGA CCA AGG GGA TAT TGA TC
PGMY09-P	G CCC AAC GGA AAC TGA TC
PGMY09-Q	CGA CCC AAG GGA AAC TGG TC
PGMY09-R	CGT CCT AAA GGA AAC TGG TC
HMB01	GCG ACC CAA TGC AAA TTG GT

^a^PGMY11 is a mix of 5 forward primers and PGMY09 is a mix of 13 reverse primers

During the first run 450 bp length DNA fragment was amplified. The reaction was carried out in 20 µl reaction mixture containing 4 mM of MgCl_2_, 200 µM of each dNTP, 0.1 µM of each PGMY primer, 1.5U of TaqNova polymerase and 4 µl of DNA. PCR cycling conditions were: initial denaturation at 94 °C for 4 min, then 40 cycles of 94 °C for 30 s, 55 °C for 45 s and 72 °C for 30 s, and final elongation at 72 °C for 4 min.

During the second PCR run 150 bp DNA fragment was amplified in a volume of 20 µl containing 3.5 mM of MgCl_2_, 200 µM of each dNTP, 0.6 µM of each GP + primer, 0.5U of TaqNova polymerase and 4 µl of the first reaction DNA product. Thermal cycling was as followed: initial denaturation at 94 °C for 4 min, then 40 cycles of 94 °C for 30 s, 40 °C for 30 s and 72 °C for 30 s, and final elongation at 72 °C for 4 min. All primers were synthesized in Genomed, Poland and the rest of PCR reagents were produced by DNA Gdansk, Poland.

Final products were separated in 2% agarose gel and visualized under UV light by SimplySafe dye (EURx, Poland). Negative (water) and positive (DNA isolated from HPV positive cervical cancer tissue) controls were added to each PCR run. For each tumour 3 independent analyses were performed and sample was classified as HPV DNA positive when at least one positive signal was observed.

### HPV genotyping assay

All samples classified as HPV DNA positive in nested PCR were genotyped by AmoyDx Human Papillomavirus (HPV) Genotyping Detection Kit (Amoy Diagnostics Co., China). This assay identifies 19 high-risk HPV DNA (16, 18, 26, 31, 33, 35, 39, 45, 51, 52, 53, 56, 58, 59, 66, 68, 70, 73, and 82) and 2 low-risk HPV DNA (6 and 11). The qPCR reaction was run in 8-tube strips with pre-loaded PCR Reaction Mixes. Each tube of the strip contained different Reaction Mix with primers and probes specific for L1 gene fragment of 2 or 3 different HPV types, hence 2 or 3 different viruses (depending on the Master Mix pre-loaded) could be detected simultaneously in one tube.

The procedure was performed according to suggestions of manufacturer and AmoyDx support. Firstly, 2.7 ul of HPV21 Enzyme Mix (containing Taq polymerase) was added to 42.3 µL of DNA sample and 5 μL of this solution was transferred into each PCR tube of 8-tube strip (in total 250 ng of DNA per reaction was added). The reaction was carried out in a volume of 30 µl using ABI7500 System (Life Technologies, USA). Thermal cycling conditions were as followed: 1 cycle of 50 °C for 2 min and 95 °C for 5 min, than 10 cycles of 95 °C for 25 s, 60 °C for 20 s and 72 °C for 20 s, and finally 31 cycles of 95 °C for 25 s, 60 °C for 35 s and 72 °C for 20 s.

Three different controls were included into each qPCR run: (1) an internal positive control designed to detect a housekeeping gene, (2) HPV^21^ positive control containing a recombinant gene with HPV plasmid DNA, and (3) no template control containing sterile water instead of DNA.

HPV types were defined based on the analysis of FAM, CY5 and VIC fluorescent signals in each well. In the cases of doubtful results analyses were repeated.

### Immunohistochemical p16 staining

P16 staining was performed using CINtec p16^INK4a^ Histology Kit (Roche, Germany). Staining procedure was performed according to manufacturer instructions. In brief, 4 µm thick sections of FFPE HNSCC tissues were deparaffinized and hydrated through a series of xylenes and alcohols. After antigen unmasking (96 °C, 10 min) and exogenous peroxidases quenching (5 min), sections were incubated with primary anti-p16 antibody (clone E6H4, RT, 30 min) followed by 30 min incubation with visualization system. P16 was visualized using DAB (3, 3′–diaminobenzidine) and for nuclear counterstaining hematoxylin was applied. Cervical cancer tissue with p16 overexpression was used as a positive control. For negative control the primary antibody was omitted.

**Fig. 1:**
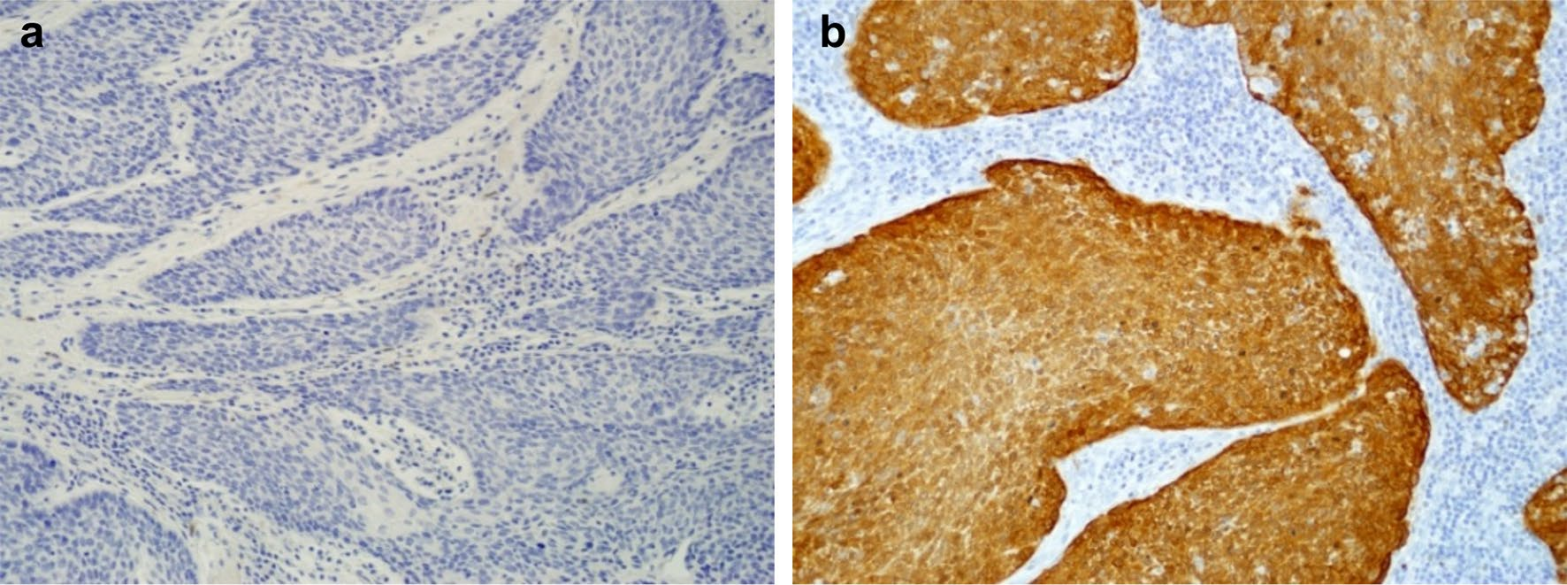
Representative images of p16 negative (**a**) and positive (**b**) signal in HNSCC tissue based on immunohistochemical staining using CINtec p16^INK4a^ Histology Kit (Roche, Germany)

Stained sections were reviewed independently by 2 researchers. Staining intensity (0—no staining, 1—weak, 2—moderate, 3—strong) and the percentage of stained tumour cells were assessed. P16 overexpression was positive if moderate or strong and diffuse, continuous (nuclear and/or cytoplasmic) staining present in > 75% of tumour cells was observed. All other staining patterns (focal, weak or no signal) were defined as p16 negative (Fig. 1).

### Determination of active HPV infection

Tumours were defined as HPV positive if they contained HPV DNA (detected during nested PCR and then confirmed by genotyping assay) and overexpressed p16 (according to immunohistochemistry). Such tissues demonstrated an active viral infection and were marked as HPV DNA + /p16 + . All other cases (HPV DNA + /p16-, HPV DNA-/p16 + and HPV DNA-/p16-) were classified as HPV negative ones.

### Statistical analysis

To determine mean and median values of continuous variables descriptive statistics were used. Associations between categorical variables were analyzed using Pearson *χ*^2^ test. To analyse prognostic potential 5-year overall survival (OS; time from the end of therapy until death from any cause within 5 years after completing the treatment) and 5-year disease free survival (DFS; time from the end of therapy until the first documented evidence of recurrent disease i.e. treatment failure, locoregional recurrence or distant metastasis within 5 years after completing the treatment) were assessed. The probabilities of OS and DFS were calculated using Kaplan–Meier method and compared by log-rank test. Because of low number of distant metastasis (*n* = 16), metastasis-free survival was not assessed. Univariate and multivariate analyses with Cox proportional regression model were carried out to select independent prognostic factors. All parameters with statistically significant influence on survival in univariate analysis were included into multivariate analysis. In all statistical analyses p-value less than 0.05 was considered significant. Calculations were performed using Statistica v.11.

We performed statistical calculations in the whole group of 155 HNSCC patients as well as in the group of 151 patients, after excluding those infected with other than HPV16 types. All analyses were also performed separately in the subgroup of 66 oropharyngeal cancer patients.

## Results

### Patients

Detailed data of 155 patients enrolled in this study were summarized in Table 1. They were between 24 and 78 years old with the mean and median age values as 56.9 and 57 years, respectively. Most of them were males (83.87%), heavy smokers (78.71%) and drinkers (56.77%). Slightly over the half of analyzed tumours were keratinizing (58.71%), in T stage 3 (50.32%), N stage 2 (54.19%) and grade II (54.84%). Most of patients (56.78%) received radiotherapy (RT)—alone or post-operative, 29.03% were treated with concurrent chemoradiotherapy (CRT) – definitive or post-operative and for 14.19% induction chemotherapy or induction chemotherapy followed by RT and/or surgery was applied. At the time of the study 39.35% of patients were alive without cancer symptoms and 23.87% of patients died from other reasons than cancer disease (mainly cardiovascular complications). Treatment failure, local recurrence and distant metastases were identified in 7, 34 and 16 patients, respectively (in total cancer progression was found in 36.77% of patients). Additionally, data of 66 patients with oropharyngeal cancer only were summarized separately in Table 2.

### HPV prevalence in HNSCC patients

**Fig. 2:**
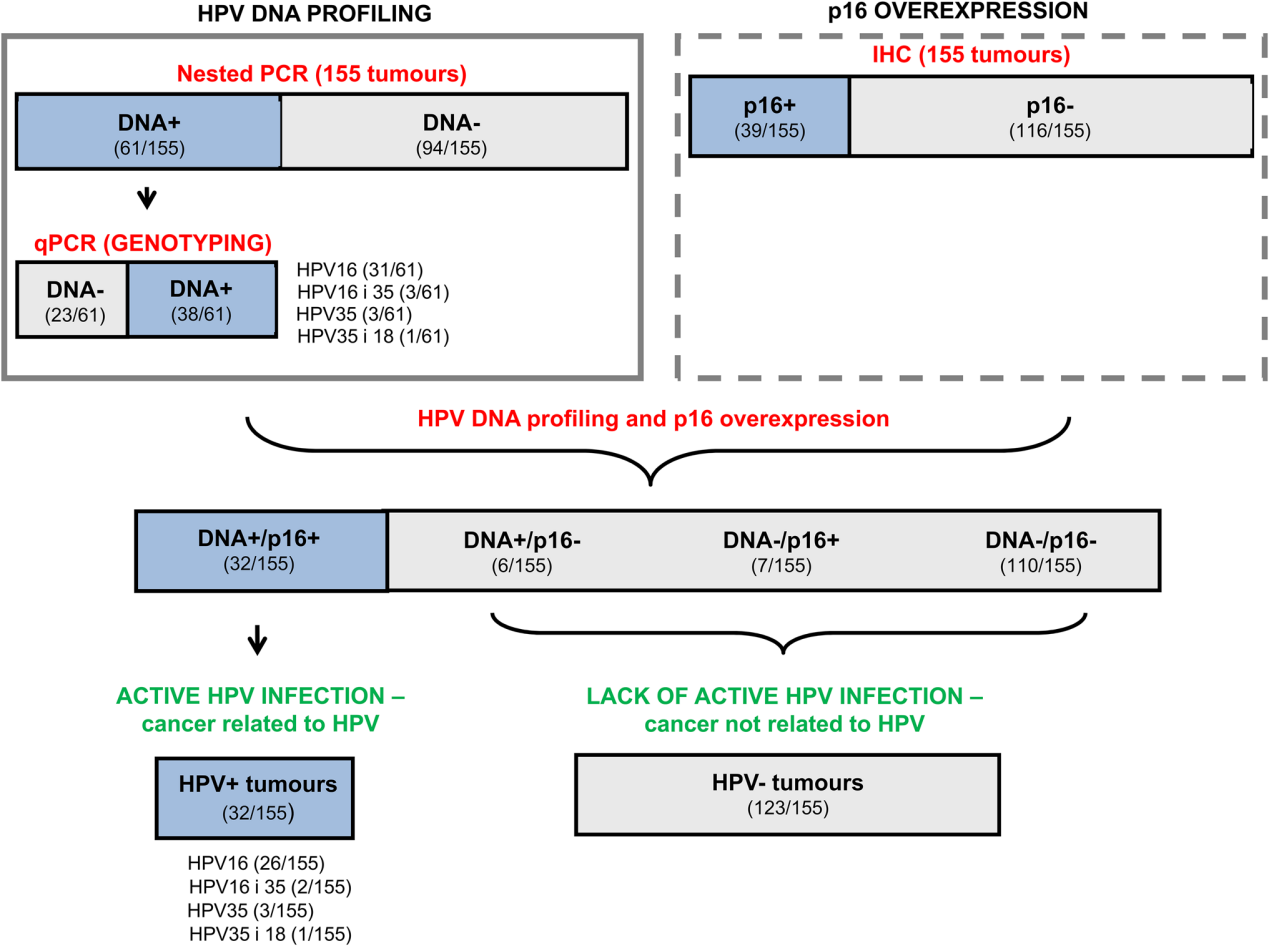
HPV prevalence in HNSCC patients from South-Central Poland. The results of nested PCR, genotyping experiments and immunohistochemical staining of p16 are presented. After combining the data of all 3 methods tumours with active HPV infection (i.e. nested PCR + /qPCR + /p16 +) were identified. Numbers of HPV positive per all analyzed cases are presented in brackets

After nested PCR analysis 61 of 155 tumours were qualified as HPV DNA positive (Fig. 2). HPV DNA positivity were verified again in qPCR analysis, where specific virus type should be identified. If there was no DNA detected during genotyping assay, we marked that tissue as HPV negative. Generally we proved HPV DNA presence in 38 (24.51%) tumours. On the other hand, p16, as the most popular surrogate marker of HPV infection, was assessed immunohistochemically and its overexpression was detected in 39 of 155 (25.16%) analyzed tumours. Combining PCR and immunohistochemical results together, we concluded that active HPV infection was detected in 20.65% (*n* = 32) of patients (Table 1). HPV16 type was the most frequent (81.25%, 26 patients) followed by HPV35 (9.38%, 3 patients) and double infections with HPV16 and 35 (6.25%, 2 patients) and HPV35 and 18 (3.12%, 1 patient).

It is worth to mention that we detected HPV DNA (in nested PCR and confirmed its presence by qPCR) in six more tissues (3.87%), but p16 was not overexpressed in them so the infection was rather transient and cancer was not related to the virus (hence we qualified those cases as HPV negative). On the other hand, 7 cases (4.52%) of HPV DNA-/p16 + were identified. All other patients (70.97%) were negative for both HPV DNA and p16 overexpression.

### HPV in relation to clinical and histopathological features

For all 155 tumours, we found statistical significant differences between HPV positive and negative tumours for most of analyzed clinical and histopathological features (Table 1). Active HPV infection was significantly more often identified in patients in a good performance status, not suffering from alcohol abuse and those with non-keratinizing tumours, contrary to HPV negative ones. HPV was found mostly (almost 85% of all cases detected) within oropharyngeal tumours, with only 1 positive tumour of larynx and none of hypopharynx identified. Moreover, tumours with active viral infection were equally distributed between smokers and non-smokers, while HPV negative tumours were much more common among heavy smokers (86.18%). Most of infected patients were treated with CRT (40.63%), while postoperative RT was the main treatment method for patients without HPV (56.92%). There was also statistical difference in treatment outcome observed between individuals with different HPV status. Most (75%) of patients with active HPV infection had no cancer symptoms at the last follow-up and only 1 case was found with treatment failure, 1 with distant metastasis and 2 with local recurrences (in total 12.51%), whereas among not infected subgroup for 43.1% of patients cancer progression was observed. We found the same statistical relations between patients with active HPV16 infection (patients with other HPV types were excluded from the analyses) and HPV negative ones. In the whole group of 155 patients we did not find any significant relations between HPV presence and patients’ age, nodal status and grade. Clinical and histopathological features in relation to active HPV infection concerning oropharyngeal cancer patients only were demonstrated in Table 2.

### Survival analysis

In the whole analyzed group of 155 patients 5-year OS and 5-year DFS were 45.61 and 61.73%, respectively. Both parameters were significantly higher in patients with active HPV infection and this was maintained also when we checked OS and DFS within oropharyngeal and non-oropharyngeal cancer patients separately. Five-year OS for patients infected with HPV was 80.89% and without infection 37.08% (*p* = 0.000). Within oropharyngeal cancer patients the probabilities of 5-year OS were 77.25 and 35.75% for HPV positive and HPV negative ones (*p* = 0.005), respectively, and for non-oropharyngeal cancer patients 100 and 37.71% (*p* = 0.007), respectively. Similarly, 5-year DFS were statistically significantly different in presented subgroups (93.0% for patients with active HPV infection and 53.35% for HPV negative ones, *p* = 0.000). Within patients with tumours of oropharynx DFS was 91.48% and 50.47% (*p* = 0.002) and with non-oropharyngeal tumours was 100 and 54.25% (*p* = 0.039) for HPV positive and HPV negative individuals, respectively.

It is also worth noting that all people (100%) infected with other than HPV16 type survived 5 years without any cancer symptoms, while HPV16 infected patients DFS was 92.05%. Five-year OS, in turn, was 75.00% for patients with non-HPV16 types and 81.68% for HPV16 positive ones.

Additionally, univariate analysis performed in the whole group of patients revealed (Table 4) that persons without active HPV infection had about four times higher probability of death and over eight times higher probability of cancer progression (treatment failure, recurrence or developing distant metastasis) than infected patients. Moreover, significantly higher OS was found for females, people in better performance status (assessed in Karnofsky scale), light smokers as well as patients with tumours of lower T stage and N stage. In turn, significantly better DFS was established for females, older people, those having tumours of lower T stage and grade as well as patients characterizing by low levels of smoking and drinking (Table 4). In the group of 66 oropharyngeal cancer patients 5-year OS depended significantly on gender, N stage, keratinization status, the level of drinking, HPV infection and treatment. On the other hand, 5-year DFS was correlated with N stage, HPV presence and treatment (Table 5).

**Table 4:** Univariate Cox proportional hazard model for 5-year overall and disease free survivals of HNSCC patients

	5-year overall survival				5-year disease free survival			
	Alive/all patients (%)^a^	HR	95% CI	*p* value^d^	Alive/all patients (%)^a^	HR	95% CI	*p* value^d^
Age								
≤ 52 years	23/51 (45.10)	1.123			27/51 (52.94)	1.451		
> 52 years	53/104 (50.96)	1.000	0.719–1.807	0.576	76/104 (73.08)	1.000	1.055–3.142	**0.028**
Gender								
Female	20/25 (80.00)	1.000			21/25 (84.00)	1.000		
Male	56/130 (43.08)	3.616	1.461–8.952	**0.001**	82/130 (63.08)	2.919	1.052–8.100	**0.019**
Performance status in the Karnofsky scale								
≤ 80%	37/90 (41.11)	1.391			58/90 (64.44)	1.216		
> 80%	39/65 (60.00)	1.000	1.026–2.625	**0.034**	45/65 (69.23)	1.000	0.729–2.230	0.385
Tumour site								
Oral cavity	11/25 (44.00)	1.232	0.700–2.422		16/25 (64.00)	1.312	0.684–3.083	
Oropharynx	36/66 (54.55)	1.000			48/66 (72.73)	1.000		
Hypopharynx	2/6 (33.33)	1.259	0.493–3.698		1/6 (16.67)	4.001	1.536–10.421	
Larynx	27/58 (46.55)	1.143	0.715–1.907	0.797	38/58 (65.52)	1.199	0.685–2.272	0.079
T stage								
1 + 2	21/29 (72.41)	1.000			25/29 (86.21)	1.000		
3 + 4	55/126 (43.65)	1.622	1.270–5.496	**0.003**	78/126 (61.90)	3.449	1.243–9.571	**0.006**
*N* stage								
0 + 1	35/60 (58.33)	1.000			41/60 (68.33)	1.000		
2 + 3	41/95 (43.16)	1.393	1.025–2.651	**0.033**	62/95 (65.26)	1.219	0.728–2.254	0.377
Grade								
1 + 2	68/133 (51.13)	1.000			91/133 (68.42)	1.000		
3	8/22 (36.36)	1.250	0.748–2.374	0.328	12/22 (54.55)	1.347	0.767–3.054	**0.013**
Keratinization								
No	35/64 (54.69)	1.000			46/64 (71.88)	1.000		
Yes	41/91 (45.05)	1.239	0.831–2.077	0.235	57/91 (62.64)	1.315	0.824–2.585	0.184
The level of smoking^b^								
≤ 200	24/33 (72.73)	1.000			28/33 (84.85)	1.000		
> 200	52/122 (42.62)	1.585	1.202–4.824	**0.007**	75/122 (61.48)	2.931	1.165–7.373	**0.012**
The level of drinking								
Low	37/67 (55.22)	1.000			50/67 (74.63)	1.000		
High	39/88 (44.32)	1.274	0.874–2.170	0.160	53/88 (60.23)	1.440	1.000–3.192	**0.043**
HPV active infection (all types)								
Present	26/32 (81.25)	1.000			30/32 (93.75)	1.000		
Absent	50/123 (40.65)	3.973	1.727–9.139	**0.000**	73/123 (59.35)	8.190	1.992–33.676	**0.000**
HPV16 active infection^c^								
Present	23/28 (82.14)	1.000			26/28 (92.86)	1.000		
Absent	50/123 (40.65)	4.333	1.723–10.894	**0.000**	73/123 (59.35)	8.015	1.795–35.786	**0.000**
Treatment								
Definitive CRT	23/31 (74.19)	1.101	0.429–2.885		25/31 (80.65)	1.123	0.349–3.744	
Surgery + CRT	7/10 (70.00)	1.000			8/10 (80.00)	1.000		
Definitive RT	5/12 (41.67)	1.530	0.673–6.740	0.079	6/12 (50.00)	3.190	0.751–13.541	0.069
Surgery + RT	33/76 (43.42)	1.547	0.697–6.984		51/76 (67.11)	1.443	0.481–6.710	
Induction CT	8/26 (30.77)	4.069	1.109–14.937		13/26 (50.00)	4.664	0.909–23.925	

*HR *hazard ratio, *CI *confidence interval

^a^Row percentage

^b^Number of cigarettes per day x years of smoking

^c^151 patients (patients infected with other than HPV16 types were excluded)

^d^Values in bold indicate statistically significant differences between groups at the *p* < 0.05 level

**Table 5:** Univariate Cox proportional hazard model for 5-year overall and disease free survivals of 66 oropharyngeal cancer patients

	5-year overall survival				5-year disease free survival			
	Alive/all patients (%)^a^	HR	95% CI	*p* value^d^	Alive/all patients (%)^a^	HR	95% CI	*p* value^d^
Age								
≤ 52 years	10/21 (47.62)	1.136			14/21 (66.67)	1.246		
> 52 years	26/45 (57.78)	1.000	0.550–2.432	0.695	34/45 (75.56)	1.000	0.513–3.422	0.555
Gender								
Female	13/15 (86.67)	1.000			12/15 (80.00)	1.000		
Male	23/51 (45.10)	4.966	1.182–20.861	**0.010**	36/51 (70.59)	1.451	0.526–6.297	0.309
Performance status in the Karnofsky scale								
≤ 80%	14/28 (50.00)	1.264			18/28 (64.29)	1.510		
> 80%	22/38 (57.89)	1.000	0.662–2.790	0.403	30/38 (78.95)	1.000	0.804–5.182	0.130
*T* stage								
1 + 2	10/15 (66.67)	1.000			13/15 (86.67)	1.000		
3 + 4	26/51 (50.98)	1.446	0.689–4.731	0.196	35/51 (63.63)	2.940	0.675–12.808	0.110
*N* stage								
0 + 1	18/24 (75.00)	1.000			21/24 (87.50)	1.000		
2 + 3	18/42 (42.86)	3.067	1.248–7.537	**0.008**	27/42 (64.29)	3.890	1.120–13.508	**0.018**
Grade								
1 + 2	33/60 (55.00)	1.000			45/60 (75.00)	1.000		
3	3/6 (50.00)	1.090	0.333–3.624	0.875	3/6 (50.00)	1.507	0.586–7.014	0.254
Keratinization								
No	19/28 (67.86)	1.000			23/28 (82.14)	1.000		
Yes	17/38 (44.74)	1.538	0.990–4.735	**0.043**	25/38 (65.79)	1.594	0.876–6.920	0.073
The level of smoking^b^								
≤ 200	16/22 (72.73)	1.000			19/22 (86.36)	1.000		
> 200	20/36 (45.45)	1.555	0.918–5.507	0.061	29/44 (65.91)	2.927	0.846–10.131	0.068
The level of drinking								
Low	21/29 (72.41)	1.000			24/29 (82.76)	1.000		
High	15/37 (40.54)	1.608	1.134–5.744	**0.017**	24/37 (64.86)	1.601	0.890–7.060	0.069
HPV active infection (all types)								
Present	21/27 (77.78)	1.000			25/27 (92.59)	1.000		
Absent	15/39 (38.46)	3.318	1.355–8.126	**0.005**	23/39 (58.97)	6.924	1.588–30.191	**0.002**
HPV16 active infection^c^								
Present	18/23 (78.26)	1.000			21/23 (91.30)	1.000		
Absent	15/39 (38.46)	3.526	1.319–9.428	**0.007**	23/39 (58.97)	6.897	1.419–33.518	**0.006**
Treatment								
Definitive CRT	16/22 (72.73)	1.057	0.407–2.762	**0.013**	20/22 (90.91)	1.000		**0.010**
Surgery + CRT	4/6 (66.67)	1.034	0.284–3.779		5/6 (83.33)	1.096	0.239–5.122	
Definitive RT	3/6 (50.00)	1.461	0.517–6.649		4/6 (66.67)	1.592	0.598–10.024	
Surgery + RT	9/13 (69.23)	1.000			10/13 (76.92)	1.288	0.430–4.581	
Induction CT	4/19 (21.05)	6.362	1.762–22.976		9/19 (47.37)	9.559	2.472–36.969	

*HR *hazard ratio, *CI *confidence interval

^a^Row percentage

^b^Number of cigarettes per day x years of smoking

^c^151 patients (patients infected with other than HPV16 types were excluded)

^ d^Values in bold indicate statistically significant differences between groups at the *p* < 0.05 level

All variables, which influenced significantly on OS and DFS in univariate analysis, were included into multivariate analysis. It revealed that in the whole group of patients independent favourable prognostic factors for OS were female gender, lower N stage and active HPV16 infection (Table 6). On the other hand, favourable prognostic factors for DFS were lower T stage and active HPV infection (overall, not only HPV16-specific) presence. It is worth to mention that in the analyzed group of patients the strongest prognostic factor for OS and DFS was HPV infection. HPV negative HNSCC patients had 4 times higher probability of death and 7.6 times higher probability of cancer progression during 5 years after treatment than infected ones.

**Table 6:** Multivariate Cox proportional hazard model

	HR	95% CI	*p* value^a^
5-year overall survival			
Gender			
Female	1.000		
Male	3.203	1.289–7.963	0.012
*N* stage			
0 + 1	1.000		
2 + 3	1.464	1.157–3.002	0.010
HPV16 active infection			
Present	1.000		
Absent	4.042	1.625–10.053	0.003
5-year disease free survival			
*T* stage			
1 + 2	1.000		
3 + 4	3.124	1.125–8.674	0.029
HPV active infection (all types)			
Present	1.000		
Absent	7.666	1.863–31.543	0.005

*HR *hazard ratio, *CI *confidence interval

^a^*p* values were examined by the Cox proportional hazard model for multivariate survival analysis

Independent favourable prognostic factors for oropharyngeal cancer patients, in turn, were: for OS -female gender, lower N stage and active HPV16 infection, and for DFS—lower N stage and HPV presence (Table 7).

**Table 7:** Multivariate Cox proportional hazard model for 66 oropharyngeal cancer patients

	HR	95% CI	*p* value^a^
5-year overall survival			
Gender			
Female	1.000		
Male	4.920	1.153–20.996	0.031
*N* stage			
0 + 1	1.000		
2 + 3	3.564	1.439–8.826	0.006
HPV16 active infection			
Present	1.000		
Absent	2.861	1.094–7.487	0.032
5-year disease free survival			
*N* stage			
0 + 1	1.000		
2 + 3	3.684	1.061–12.792	0.040
HPV active infection (all types)			
Present	1.000		
Absent	6.647	1.524–28.986	0.012

*HR *hazard ratio, *CI* confidence interval

^a^*p* values were examined by the Cox proportional hazard model for multivariate survival analysis

## Discussion

More and more researchers have been joining the discussion about the role of HPV in head and neck carcinogenesis recently, however, the number of publications concerning prevalence of active infection in HNSCC patients are still in minority. Ndiaye et al. (2014) performed meta-analysis including 148 studies (12,163 cases from 44 countries) and they assessed the prevalence of active HPV infection based on simultaneous detection of HPV DNA and p16 overexpression (similarly as we did). The active form of infection was present in 6.8, 39.7 and 19.1% of oral cavity, oropharyngeal and laryngeal cancer cases, respectively, while we identified 16.0, 40.9 and 1.7% of positive cases, respectively. Only HPV prevalence within oropharynx was similar. Differences noted in other two localizations could be probably a result of geographical differences and high heterogeneity in methodology used by us and studies included into meta-analysis. Even if the same biomarkers were used (HPV DNA and p16) to assess HPV presence, the selection of experimental assay and interpretation of staining in different laboratories may lead to highly different final results Castellsagué et al. (2016) in a second recent international study (3680 samples from 29 countries) assessed the virus prevalence based on simultaneous presence of HPV DNA, E6*l mRNA and p16 overexpression. They estimated that globally HPV was present in 3.0%, 18.5% and 1.5% of oral cavity, oropharyngeal and laryngeal tumours, respectively, and for Europe these percentages were 2.1%, 15.9% and 1.2%, respectively. The worldwide as well as European HPV rates for oropharyngeal and oral cavity cancers were much lower than we found. It may be the effect of looking for three biomarkers simultaneously in one tissue as well as other aspects of methodology, geographic location or patients qualification criteria. However, HPV prevalence within laryngeal cancer patients is quite similar to obtained by us.

In Poland the data concerning HPV infection rate among patients with HNSCC remain largely inconsistent, ranging from 0 (Golusinski et al. 2012) to 57% (Mazurek et al. 2016) (we identified 20.65% of HPV positive cases in this study). This depends mainly on the tumour site, geographical region and especially method used for HPV detection. Detailed data on HPV prevalence in Polish HNSCC patients are summarized in Table 8. To the best of our knowledge, there was only one Polish study where active HPV infection was assessed so far, but no case of viral infection was detected in analyzed group of patients (Golusinski et al. 2012). Therefore, in the present work active HPV infection in HNSCC patients was detected for the first time in our country. In the present study the prevalence of HPV depended on tumour site. We detected active HPV infection in 40.9% of oropharyngeal SCC patients. It was lower than in the study described by Mazurek et al. (2016) but much higher than in papers of Szkaradkiewicz et al. (2002) and Polz-Gruszka et al. (2015)—57.0, 10.71 and 26.7% of HPV positive tumours, respectively. On the other hand, we found HPV infection in only 16.0% of oral cavity tumours, that is half less than Prawdzic Seńkowska et al. (2019) identified. We also detected low HPV prevalence among laryngeal SCC patients (1.7%), what is in agreement with results obtained by Snietura et al. (2011), who have not identified any HPV within this localization. On the other hand, our results are in opposite to Józefowicz-Korczyńska et al. (2014), Morshed (2010) and Polz-Gruszka et al. (2015) who found 23.0, 27.7–35.5 (depending on the method used) and 36.0% of HPV positive laryngeal tumours, respectively. It is important to note that most of presented results might be overstated because of detection only HPV DNA and not active viral infection.

**Table 8:** A review of the literature concerning HPV prevalence in Polish patients with squamous cell carcinoma of head and neck

Authors	No. of specimens	Tumour site	Method/kit used for HPV detection	HPV prevalence [%]	Genotypes detected^a^	Active infection assessed (yes/no)
Szkaradkiewicz et al. (2002)	28	Oropharynx	PCR-ELISA	10.7	–	No
Morshed (2010)	130	Larynx	Immunohistochemistry	27.7	–	No
	93		SPF-10 PCR and DNA enzyme immunoassay	35.5		
Polz et al. (2010)	60	Oral cavity oropharynx	INNO-LiPA HPV genotyping CE amp kit	25.0	HPV16 (87%) not identified types (13%)	No
Snietura et al. (2011)	66	Oral cavity/ oropharynx	Real time high risk HPV test for detection of 14 HPV types	6.9 (13.6% within oral cavity/ oropharynx and 0% within larynx)	HPV16 (100%)	No
	65	Larynx				
Golusinski et al. (2012)	50	Oral cavity oropharynx larynx	p16 immunostaining and GP5 + /6 + PCR, followed by RLB hybridization	0.0	none	Yes
Józefowicz-Korczyńska et al. (2014)	100	Larynx	INNO-LiPA HPV genotyping extra assay	23.0	HPV18 (30%) HPV16 (22%) not identified types (48%)	No
Polz-Gruszka D. et al. (2015)	50	Larynx	INNO-LiPA HPV genotyping extra assay	32.5 (26.7% within oropharynx and 36.0% within larynx)	HPV16 (69%) HPV45, 59 and 68 (31%)	No
	30	Oropharynx				
Mazurek et al. (2016)	63	Oropharynx	qPCR (assessment of HPV16 DNA in plasma and tumour samples)	38.0 in plasma samples 57.0 in tumour samples	only HPV16 was identifying	No
Prawdzic Seńkowska et al. (2019)	47	Oral cavity	GenoFlow HPV array test kit for detection of 33 HPV types	31.9	HPV16 (47%) HPV 18 (7%) HPV 43/44 (40%)	No

^a^In brackets percentages per all detected HPV positive cases are presented

The most frequent virus type among Polish HNSCC patients was HPV16, but HPV 18, 43/44, 45, 59 and 68 were also detected (Table 8). In our group, beside HPV16 and 18, active infection of HPV35 was identified—for the first time in Polish HNSCC patients. Interestingly, all viruses other than HPV16 (independently if it was single or double infection) were identified within oropharyngeal tumours, which may suggest that this localization is more prone to the infection with non-HPV16 types as well as multiple HPV infections. Most frequent virus type worldwide was HPV16 (about 80% of all cases) followed by few cases of HPV6, 18, 33, 35 and other even less frequent (Ndiaye et al. 2014; Castellsagué et al. 2016). HPV16 was also the most commonly detected type in our study (81.25% of HPV positive cases). Additionally, we identified 6 (3.9%) tumours with HPV35 infection (3 with single HPV35 infection, 2 with HPV35 and 16 double infection and 1 with HPV35 and 18 double infection). Castellsagué et al. (2016) identified 0.3% of patients with HPV35 and it was single infection. Similarly, Ndiaye et al. (2014) estimated 0.1% of HPV35 positive cases in Europe and 0.2% worldwide within oropharyngeal tumours (in oral cavity and laryngeal tumours this type it was even rarer—outside the six most common HPV types). Our results may suggest that HPV35 has an important role in oropharyngeal carcinogenesis in South-Central Poland region—contrary to other Polish regions, Europe and the rest of the world, however, this hypothesis need a confirmation in much larger group of Polish patients.

The other important topic at the present study is an association between HPV status and clinical, epidemiological and histopathological characteristics of HNSCC patients. A review of the literature indicates that the data on it are highly conflicting. It was shown that HPV positivity may be associated with female sex (Tsai et al. 2019), white race, better performance status (Fakhry et al. 2008), younger age of patients, college education, marihuana smoking (Gillison et al. 2008), low level of tobacco use and low alcohol consumption (Józefowicz-Korczyńska et al. 2014) as well as contrary—high levels of smoking and drinking (Meng et al. 2018), and higher response rates after treatment (Fakhry et al. 2008). Tumours with viral infection were more likely to have higher T stage, be poorly differentiated and have basaloid features (Fakhry et al. 2008). There were also some authors who found no statistically significant association between the presence of HPV, epidemiological and clinicopathological features (Morshed 2010). It seems reasonable that all these conflicting data result in part from the differences in the number of analyzed subgroups and their heterogeneity in terms of clinical and histopathological features.

In the present study, in the whole group of 155 patients as well as in the subgroup of oropharyngeal cancer patients, we have shown that patients having active HPV infection (positivity of qPCR HPV analysis + overexpression of p16 in IHC staining) have better prognosis. We found statistically significant differences in 5-year OS and DFS when we were comparing HPV positive and negative cases in the whole group of HNSCC patients as well as within oropharyngeal and non-oropharyngeal cancer patients separately. Similar results were obtained by Fakhry et al. (2017) who calculated that 5-year OS were 78.6 and 43.0% for patients with HPV positive and negative oropharyngeal SCC, respectively. Surprisingly, we also found, to the best of our knowledge for the first time, 100% 5-year DFS for HNSCC patients infected with virus types other than HPV16 (in our study HPV35 or HPV35 simultaneously with HPV18 were detected in those cases). HPV type seems then influencing the patient’s survival. However, this hypothesis need a confirmation in much larger group of patients with different HPV types including HPV35.

There is also a global discussion about good prognosis of patients with head and neck tumours related to HPV. However, the mechanism of better survival of those patients remains unclear. In the literature authors consider 4 possible reasons explaining this phenomenon: (1) an increase in radio- and/or chemosensitivity (Mirghani et al. 2015; Ziemann et al. 2015), (2) stimulation of immune response (Lechien et al. 2019), (3) more favourable epidemiological, clinical and histopathological features (Pan et al. 2018), and (4) absence/low level of gene mutations in HPV positive tumours. It was demonstrated (Stransky et al. 2011; Agrawal et al. 2011) that HPV positive tumours accumulated far fewer mutations than HPV negative ones especially within *TP53, CDKN2A, PTEN, PIK3CA, FBXW7, HRAS* and *NOTCH1* genes, what influenced the functioning of key cellular signalling pathways including EGFR/PI3K/AKT/mTOR. All mentioned hypotheses are likely to be possible, hence further investigations are highly needed.

Generally, better survival of HPV positive oropharyngeal cancer patients comparing to HPV negative ones has been well established (Fakhry et al. 2017; D’Souza 2016; Meng et al. 2018). However, the data concerning prognoses of non-oropharyngeal cancer patients are still conflicting. Most authors (Fakhry et al. 2017; Hernandez et al. 2014; Morshed 2010; Xu et al. 2014) have not shown any statistically significant differences in survival of patients with tumours outside the oropharynx according to their HPV status, although there were also some studies which demonstrated better (Wookey et al. 2019) or contrary—poorer (Duray et al. 2012) prognosis for HPV-related tumours. Interestingly, Salazar et al. (2014) have noted that to identify HPV-associated non-oropharyngeal HNSCC with better prognosis both p16 and HPV DNA assessing were necessary (a single test was not enough). Similarly, D’Souza (2016) suggested that p16 and HPV PCR likely do not have prognostic potential when used alone among non-oropharyngeal SCC, however cases with both p16 and HPV DNA positivity have (not significantly but still) improved survival.

In the present study multivariate analysis performed in the whole group of patients revealed that female gender, lower N stage and active HPV16 infection were favourable independent prognostic factors for OS, and lower T stage and HPV infection for DFS. In the analyzed group of HNSCC patients (all localizations) the presence of HPV was the strongest favourable independent prognostic factor (and the only one to be repeated) for both OS and DFS. The risk of death and cancer progression 5 years after treatment were over 4 and over 7.6 times higher (*p* = 0.003 and *p* = 0.005, respectively) in the group of not infected patients comparing to those with active HPV. Many researchers get similar results. Fakhry et al. (2017) demonstrated that within patients with oropharyngeal SCC the risk of death was lower for women than men (HR = 0.55; *P* = 0.04) even after the tumour HPV status has been taken into account. In contrast, for non-oropharyngeal SCC, HPV positivity and sex were not associated with OS. We noted the risk of death for men 3.2 times higher than for women (*p* = 0.012) in the group of 155 patients and 4.9 times (*p* = 0.031) among oropharyngeal cancer patients only. D’Souza (2016), in turn, noted that the risk of mortality was 75% lower for HPV-related oropharyngeal SCC compared to HPV-unrelated (HR = 0.25, 95% CI = 0.18–0.34). In contrast, among non-oropharyngeal SCC patients active HPV infection was not a significant predictor of survival. Additionally, contrary to us, they found that alcohol use was significant independent predictor of non-oropharyngeal SCC survival.

Taking into account favourable prognosis of some HPV positive HNSCC patients, there have been many clinical studies ongoing, in which different strategies of treatment de-escalation have been tested (for review see—Mirghani and Blanchard 2018), however decreasing the treatment intensification would lead to the potential risk of reducing treatment efficacy. Hence, there is a need of great caution in planning and clinical implementation of such strategies, because it was shown that in the group of HPV positive HNSCC patients there are some with worse prognoses. Biesaga et al. (2018) showed that patients with lower HPV16 load (the number of HPV16 genome copies per cell) had lower OS and DFS. Yoo et al. (2019), in turn, proved that non-oropharyngeal cancer, poor performance status and presence of HPV18 were independent poor prognostic factors in patients with HPV positive HNSCC and Tinhofer et al. (2015) noticed that in the group of HPV positive oropharyngeal cancer patients current smokers had worse 2-year survival rates compared to never/former smokers. Individuals with mentioned risk factors might not be candidates for de-escalation treatment.

## Conclusions

In this study, we identified high prevalence of active HPV infection in HNSCC patients from South-Central Poland, especially within oropharyngeal tumours. HPV16 type was the most frequent, followed by HPV35 and HPV18. Interestingly, active infection of HPV35 was identified for the first time in Polish HNSCC patients and our results suggest that HPV35 may have an important role in oropharyngeal carcinogenesis in the South-Central Poland region, contrary to other Polish regions, Europe and the rest of world, where this type prevalence is very low.

Our results clearly showed that the presence of active HPV infection in analyzed group improved survival of both oropharyngeal and non-oropharyngeal cancer patients. To the best of our knowledge, this is the first time when 100% 5-year DFS for HNSCC patients infected with HPV other than HPV16 type was reported.

Multivariate analysis revealed that (in the analyzed group of 155 HNSCC patients) female gender, lower N stage and active HPV16 infection were favourable independent prognostic factors for OS and lower T stage and HPV infection for DFS. In the whole group of patients the presence of active HPV infection was the strongest favourable independent prognostic factor for both OS and DFS. The risk of death and cancer progression 5 years after treatment were much higher in the group of HPV negative patients comparing to infected ones (similar results were obtained also for oropharyngeal cancer patients). Hence, it seems reasonable that presence of active HPV infection should be taken into account during treatment planning.
